# Inertial sensors for gait monitoring and design of adaptive controllers for exoskeletons after stroke: a feasibility study

**DOI:** 10.3389/fbioe.2023.1208561

**Published:** 2023-08-07

**Authors:** Jesús De Miguel-Fernández, Miguel Salazar-Del Rio, Marta Rey-Prieto, Cristina Bayón, Lluis Guirao-Cano, Josep M. Font-Llagunes, Joan Lobo-Prat

**Affiliations:** ^1^ Biomechanical Engineering Lab, Department of Mechanical Engineering and Research Centre for Biomedical Engineering, Universitat Politècnica de Catalunya, Barcelona, Spain; ^2^ Institut de Recerca Sant Joan de Déu, Esplugues de Llobregat, Spain; ^3^ Department of Biomechanical Engineering, University of Twente, Enschede, Netherlands; ^4^ Hospital Universitari Múttua de Terrassa, Barcelona, Spain; ^5^ ABLE Human Motion, Barcelona, Spain

**Keywords:** stroke, wearable sensors, inertial sensors, IMU, gait analysis, gait assessment, rehabilitation, exoskeleton

## Abstract

**Introduction:** Tuning the control parameters is one of the main challenges in robotic gait therapy. Control strategies that vary the control parameters based on the user’s performance are still scarce and do not exploit the potential of using spatiotemporal metrics. The goal of this study was to validate the feasibility of using shank-worn Inertial Measurement Units (IMUs) for clinical gait analysis after stroke and evaluate their preliminary applicability in designing an automatic and adaptive controller for a knee exoskeleton (ABLE-KS).

**Methods:** First, we estimated the temporal (i.e., stride time, stance, and swing duration) and spatial (i.e., stride length, maximum vertical displacement, foot clearance, and circumduction) metrics in six post-stroke participants while walking on a treadmill and overground and compared these estimates with data from an optical motion tracking system. Next, we analyzed the relationships between the IMU-estimated metrics and an exoskeleton control parameter related to the peak knee flexion torque. Finally, we trained two machine learning algorithms, i.e., linear regression and neural network, to model the relationship between the exoskeleton torque and maximum vertical displacement, which was the metric that showed the strongest correlations with the data from the optical system [*r* = 0.84; ICC(A,1) = 0.73; ICC(C,1) = 0.81] and peak knee flexion torque (*r* = 0.957).

**Results:** Offline validation of both neural network and linear regression models showed good predictions (*R^2^* = 0.70–0.80; MAE = 0.48–0.58 Nm) of the peak torque based on the maximum vertical displacement metric for the participants with better gait function, i.e., gait speed > 0.7 m/s. For the participants with worse gait function, both models failed to provide good predictions (*R^2^* = 0.00–0.19; MAE = 1.15–1.29 Nm) of the peak torque despite having a moderate-to-strong correlation between the spatiotemporal metric and control parameter.

**Discussion:** Our preliminary results indicate that the stride-by-stride estimations of shank-worn IMUs show potential to design automatic and adaptive exoskeleton control strategies for people with moderate impairments in gait function due to stroke.

## 1 Introduction

One of the main challenges of robotic devices for post-stroke gait rehabilitation is the tuning process of the control parameters (Fricke et al., 2020a; Labruyère, 2022; Melendez-Calderon and Maggioni, 2022; van Dellen and Labruyère, 2022). There is a lack of systematic or automatic procedures to help clinicians in the selection of appropriate parameter values depending on the selected task and the level of impairment (Morris et al., 2023). In general, therapists manually tune the control parameters, especially the assistance level, based on the visual assessment of the patient, and they are often kept constant throughout the whole session or treatment, which might lead to reduced training effect (Park et al., 2019; van Dellen and Labruyère, 2022). Previous studies have shown that automatic tuning of this assistance might be more optimal than manual tuning (Fricke et al., 2020a).

The majority of the control strategies implemented in exoskeletons for post-stroke rehabilitation provide assistance based on the error between the actual and reference positions (Fricke et al., 2020a; de Miguel-Fernández et al., 2023a; Morris et al., 2023). In this way, the robotic assistance can be tailored on the basis of the user’s performance. An alternative to these control strategies based on predefined joint references might be to adapt the assistance based on spatiotemporal metrics, e.g., stance duration, stride length, or foot clearance. This approach has the main benefit of using metrics that are good biomechanical descriptors of the level of gait impairment post stroke and have a strong relationship with the exoskeleton control parameters (Sulzer et al., 2009; Koopman et al., 2013; Mizukami et al., 2018; Fricke et al., 2020b; Pan et al., 2022). Therefore, these adaptive controllers based on spatiotemporal metrics can also be useful to complement or guide the automatic tuning of the control parameters.

In previous years, gait monitoring algorithms based on Inertial Measurement Units (IMUs) have become very popular as a method to estimate spatiotemporal metrics. IMU-based systems are portable and of low cost when compared to traditional stationary systems, i.e., marker-based optical motion tracking, instrumented treadmills, or pressure-sensitive walkways. Several studies have shown that it is possible to obtain reliable estimations of average gait spatiotemporal metrics of post-stroke individuals using IMU sensors, yet little is known regarding their reliability in estimating spatiotemporal metrics for each stride (Yang et al., 2013; Parisi et al., 2016; Visi et al., 2017; Wang et al., 2018; Feuvrier et al., 2020; Feuvrier et al., 2020; Arens et al., 2021; Arumukhom Revi et al., 2021; Laidig et al., 2021; Romijnders et al., 2021; Hendriks et al., 2022). Thus, it is still unclear whether spatiotemporal parameters estimated with IMUs can be used to automatically adapt the control parameters of an exoskeleton for post-stroke gait rehabilitation.

In this work, we evaluate the use of shank-worn IMUs for gait monitoring after stroke and for developing an automatic and adaptive method to tune the assistance provided by a unilateral knee-powered exoskeleton (ABLE-KS), i.e., adapting the control parameter related to the peak knee flexion torque. Specifically, we sought an answer to the following question: *can shank-worn IMUs be used to design adaptive control strategies for knee exoskeletons for post-stroke rehabilitation?*

To answer the posed question, first, we addressed the following points: (i) evaluation of the reliability of estimating gait spatiotemporal metrics using shank-worn IMUs after stroke; (ii) evaluation of the relationship between the parameter that controls the peak knee flexion torque and biomechanical metrics estimated with shank-worn IMUs.

## 2 Methods

### 2.1 Experimental protocol

**FIGURE 1:**
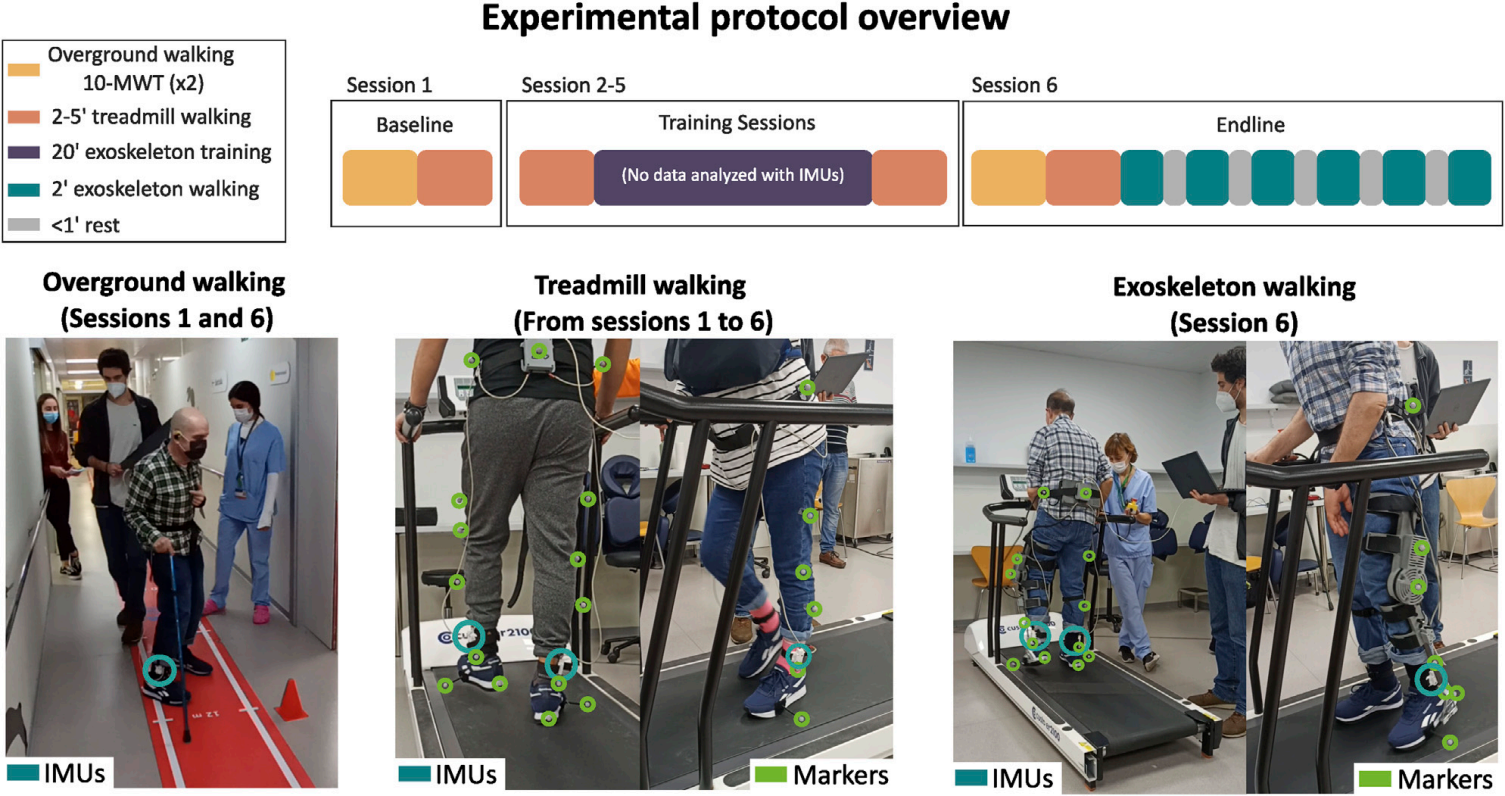
Experimental protocol overview. The experimental protocol included six sessions, namely, baseline (session 1), training (sessions 2–5), and endline (session 6) sessions. Treadmill walking was carried out in each session, overground walking, i.e., the 10-Meter Walk Test, was carried out only in the baseline and endline sessions, and walking with the exoskeleton to analyze the relationship between peak knee flexion torque and biomechanical metrics was carried out only in the endline session. Note that the data from the training sessions (indicated in purple), in which the participants walked with the exoskeleton on the treadmill, were not used for the present study.

Six post-stroke participants were recruited at the Hospital Universitari Mútua de Terrassa (Barcelona, Spain) to be involved in an observational study approved by the Medical Research Ethical Committee (MREC) of the Hospital Universitari Mútua de Terrassa under the number: E/22-082/S1. The clinical trial was carried out at the same hospital in December 2022. The experimental protocol (see Figure 1) consisted of six sessions with three different objectives: (1) comparing the performance of the IMU-based system against an optical motion capture system while walking on a treadmill (sessions 1–6); (2) validating the IMU-based system while walking overground performing the 10-Meter Walk Test (10MWT; sessions 1 and 6); and (3) investigating the relationship between the parameter that controls the peak knee flexion torque provided with a knee exoskeleton (i.e., ABLE-KS) and spatiotemporal metrics obtained with the IMU-based system (session 6).

For sessions 1–6, the participants were asked to walk on a treadmill (er2100, custo med GmbH, Germany) for up to 5 min at a comfortable speed, which remained constant during the whole session. Kinematic data were collected at 120 Hz with an optical motion capture system (V120:Trio, OptiTrack, NaturalPoint Inc., Corvallis, OR, USA). Reflective markers were placed on the sacrum and bilaterally on the iliac crests, midpoint of the thigh segments, lateral femur condyles, midpoint of the shank segments, lateral malleoli, fifth metatarsal heads, and posterior surface of the calcanei.

In sessions 1 and 6, the participants also performed the 10MWT twice per session, which was used to validate the IMU-based system while walking overground. The walking speeds estimated with the IMUs were compared with the ones measured by the experimenters with a digital stopwatch.

For the endline session, the participants were asked to walk on the treadmill with the ABLE-KS knee exoskeleton providing different levels of assistance during the swing; the peak knee flexion torque was set to 0, 1, 2, 3, and 4 Nm. The participants walked for 2 min on each torque condition starting from 0 Nm to 4 Nm. Apart from providing knee flexion assistance, the exoskeleton also provided knee stability assistance during stance and knee extension assistance at the end of the swing phase. All the control parameters were kept constant for all the conditions, with the exception of the peak knee flexion torque.

### 2.2 Participants

Suitable candidates were identified as individuals capable of performing independent gait and exhibiting mild to moderate gait deviations due to stroke (Salbach et al., 2004). Individuals were eligible for inclusion if they met the following criteria: (1) age above 18 years, (2) unilateral ischemic or hemorrhagic chronic (≥6 months) stroke, (3) Functional Ambulation Categories (FAC) score ≥2, and (4) comfortable treadmill walking speed ≥0.5 km/h. The exclusion criteria included: (1) high levels of spasticity of muscle tone (resistance to passive movement), as represented by the modified Ashworth scale scores of ≥3, (2) premorbid disability of the lower extremity, (3) skin problems or ongoing infections in areas in contact with the exoskeleton, (4) impaired cognition, (5) relevant comorbidities (e.g., chronic heart failure, uncontrolled diabetes or hypertension, chronic obstructive pulmonary disease, medical or family history of osteoporosis, or a history of fragility fractures in the last 2 years), and (6) pregnancy or breastfeeding.

In total, six participants with left-side hemiplegia due to stroke were enrolled for this study (see Table 1). All participants provided informed consent before starting the study.

**TABLE 1:** Study participants’ characteristics.

Participant	Age (years)	Body mass (kg)	Height (cm)	Gender	Type of stroke	Chronicity (years)	Regular assistive device	FAC	MAS knee Ext/Flex	10MWT (m/s)	Treadmill speed (km/h)
ID 1	71	64	165	Male	Ischemic	11.0	None	4	0/1	1.14	1.3
ID 2	56	62	150	Female	Ischemic	0.5	Cane and AFO	2	0/0	0.34	0.5
ID 3	68	66	169	Male	Hemorrhagic	12.0	Cane and AFO	4	0/1	0.69	0.8
ID 4	58	56	156	Female	Hemorrhagic	30.0	Cane	3	0/1	0.55	1.0
ID 5	40	85	169	Male	Ischemic	0.5	AFO	4	0/0	0.93	1.5
ID 6	58	61	154	Male	Ischemic	7.0	Cane	4	1/2	0.48	0.9

AFO, ankle–foot orthosis; FAC, Functional Ambulation Category; 10MWT, 10-Meter Walk Test.

### 2.3 Experimental setup

The ABLE-KS is a wearable, unilateral, powered knee exoskeleton that provides knee stability assistance during stance, and flexion and extension assistance during swing. Further details about the knee exoskeleton can be found in de Miguel-Fernández et al. (2023b). The synchronization with the user is based on the detection of the foot–ground contacts of both legs following a threshold-based algorithm that uses shank angle and velocity.

Kinematic shank data were obtained by means of two IMUs (BNO055, Bosch, Germany) attached to both shanks at the position closest to the ankle (see Figure 1). Raw linear accelerations (±4 g range) and angular velocities (±2,000 deg/s range) were measured in the three sensor local axes at a sampling frequency of 100 Hz. The IMUs were connected to a microcontroller (Teensy 4.0; PJRC, Sherwood, OR, USA) via an i2C protocol connection to store and process data.

As described previously, the participants were asked to walk with the ABLE-KS with five different peak knee flexion torques (i.e., 0, 1, 2, 3, and 4 Nm). Note that during sessions 2–5, the participants had already trained with the different torque levels.

### 2.4 Gait monitoring with shank-worn IMUs after stroke

**FIGURE 2:**
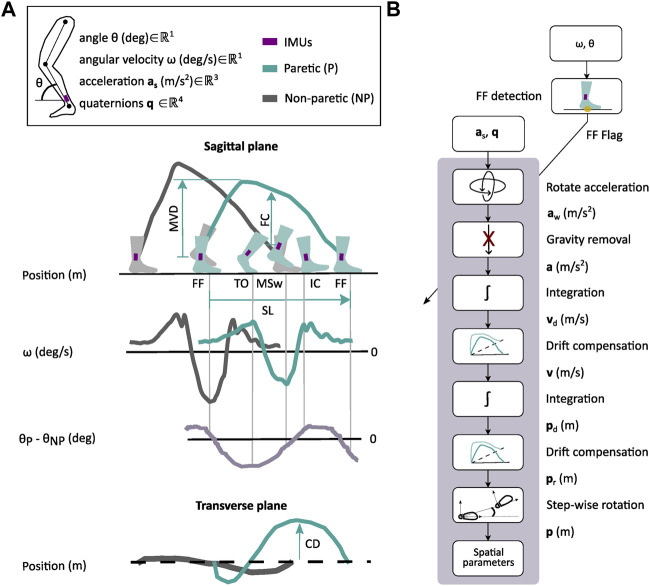
Gait monitoring with shank-worn IMUs. **(A)** Gait events, i.e., flat-foot (FF), toe-off (TO), mid-swing (MSw), and initial contact (IC), are detected on the basis of the angular rates (*ω*) of the paretic (P) and non-paretic (NP) shanks and the difference between angular displacements (*θ*). The sagittal plane projection of the estimated IMU trajectory is used to estimate the maximum vertical displacement (MVD), foot clearance (FC), and stride length (SL). Circumduction (CD) is extracted from the transverse plane projection of the IMU trajectory, i.e., maximum lateral displacement. **(B)** General algorithm pipeline to estimate spatial parameters. The algorithm runs every time that a new FF event is detected by using *ω* and *θ*. For each period of interest, the acceleration signal (**a**_**s**_) is transformed to a fixed world frame (**a**_**w**_) and gravity is removed. The resulting acceleration signal (**a**) is integrated twice to get the velocity (**v**_**d**_) and position (**p_d_**) of the IMU. From the computed trajectories, the drift is removed (**v**, **p**_**r**_), and the position is rotated for every step (**p**) to get the spatial parameters.

The algorithm implemented in the current study to monitor spatiotemporal metrics using IMUs was similar to others found in the literature (Arens et al., 2021; Laidig et al., 2021; Uchitomi et al., 2022). First, the gait events of interest, i.e., initial contact (IC), flat-foot (FF), toe-off (TO), and mid-swing (MSw), were detected by using the angle and velocity of the paretic and non-paretic shanks (see Figure 2A). Precise detection of gait events in the present algorithm is critical to accurately estimate the temporal and spatial metrics. Second, we estimated the sensor linear displacement by double-integrating the acceleration data during the swing phase (see Figure 2B). Finally, temporal metrics were defined using the phases limited by the detected gait events, and the spatial metrics were obtained from the estimated 3D trajectory (see Figure 2). For the spatial metrics, synchronized reset and initialization of the acceleration integrators were required to get an accurate estimation.

To detect the gait events of interest, i.e., IC, FF, TO, and MSw, we used a threshold-based algorithm that fuses the shanks' angular kinematics, i.e., angle and velocity, and time elapsed between two consecutive events (see Figure 2A), which we have previously validated in participants after stroke (de Miguel-Fernández et al., 2022). We combined the shank angle of the paretic and non-paretic sides to increase the robustness in the detection of gait events in pathological gait (Arens et al., 2021; Laidig et al., 2021). Each gait event was associated with three independent detection parameters: (1) angular velocity threshold to detect local maxima or minima, (2) angle difference threshold between shanks to consider the detection of the event, and (3) minimum time required between events to avoid false positives. All the participants shared the same angular velocity threshold of 100 deg/s and a time threshold of 0.5 s. The angle difference between shanks ranged from 5 to 15 degrees, depending on the step length of each participant, i.e., lower step length was associated with a lower angle threshold. These target levels were chosen on the basis of pilot tests performed with individuals after stroke. The thresholds of the gait event detection algorithm were confirmed to be appropriate based on real-time data, i.e., shank angular kinematics and event detection flags.

Both IC and TO were detected by estimating local maxima in the angular velocity of the shank (see Figure 2A). We used the difference in the angle between both shanks to differentiate between events. We classified the events as IC when the angular velocity had a local maxima and the angle difference was positive, while the TO was classified when the angle difference was negative. For the MSw, we identified the local minima in the angular velocity of the same leg with a difference in the angle between both shanks below an angle threshold. The foot was assumed to be fully flat on the ground (FF) when the MSw event of the contralateral leg occurred, as described by Uchitomi et al. (2022). Note that FF events are commonly detected by using the magnitude of the acceleration vector measured by the IMUs (Pierleoni et al., 2019), although it has been shown that angular velocity–based algorithms perform significantly better than acceleration-based algorithms, especially for pathological gait (Arens et al., 2021; Laidig et al., 2021; Uchitomi et al., 2022).

Every time an FF event occurred, the three-axis linear accelerations in the local sensor frame were transformed into the world frame using the rotation matrix obtained from the IMU, i.e., unit quaternions computed directly by the onboard Kalman filter (see Figure 2B). Then, the gravity term was removed from the acceleration in the vertical axis. The resulting acceleration signals were integrated twice using trapezoidal integration to obtain the 3D displacement of the IMU. After the first integration of the acceleration, the velocity drift was removed, assuming that the velocity at the past and current FF events was zero. To correct the position drift, we set the three components of the position to zero at the previous FF event, and only the vertical component was set to zero at the current FF event. Finally, the 3D IMU position trajectories of each step were rotated to start with the same orientation.

The stride time was estimated as the time between two consecutive IC events of the same leg. Stance time, as a percentage of the gait cycle, was estimated as the time between the first IC and TO with respect to the total stride time of the same leg. Swing time was approximated as the time between the TO and IC of the next gait cycle with respect to the total stride time of the same leg.

The stride length (SL) was defined as the maximum distance in the anterior–posterior direction measured between two successive stationary periods (see Figure 2A). Circumduction (CD) was defined as the maximum lateral displacement of the IMU during the swing phase (see IMU trajectory projected to the transverse plane in Figure 2A). Foot clearance (FC) was defined as the vertical position of the 3D IMU trajectory at the MSw. The maximum vertical displacement (MVD) was defined as the maximum vertical position of the IMU during the swing phase. The spatiotemporal metrics obtained from the motion capture system, which were used as ground truth, were computed in the same way using the markers placed at the ankle, heel, and toe. Finally, the gait speed during the 10MWT was computed as the average stride speed of both paretic and non-paretic sides, which was calculated by dividing each stride length by each stride time.

### 2.5 Regression-based automatic and adaptive controller

We developed a control scheme based on regression models to automatically adapt the exoskeleton peak knee flexion torque parameter based on a spatiotemporal metric estimated from the IMUs data (Figure 6A). The spatiotemporal metric that was used as input for the adaptive controller was the metric that presented the strongest correlation when compared with the optical system and also presented a high correlation with the peak knee flexion torque parameter. Specifically, the proposed controller follows these steps: 1) the spatiotemporal metric is estimated from the previous strides using the data from IMUs; 2) the estimated spatiotemporal metric is compared to the desired value (set by the therapist), and the variation is fed to a regression model; and 3) the regression model generates a new value for the peak knee flexion torque parameter that is used by the torque profile generator to set the device torque for the next step.

Two types of regression models were evaluated offline: 1) linear regression model and 2) neural network. Both models were trained with 80% of the available steps of each participant for each condition to model the relationship between the variations of the selected spatiotemporal metric and peak knee flexion torque parameter. The remaining 20% of the steps of each participant were used to validate the model.

### 2.6 Outcomes and statistical analysis

To compare the estimation for the metrics presented in Section 2.4 with the ground truth values obtained with the optical motion capture system, we used the mean absolute error (MAE), Pearson’s correlation coefficient (*r*), and inter-rater reliability based on intra-class correlation analysis (ICC). The MAE was used as a measure of statistical dispersion of the error, while the ICC evaluated the agreement ICC(A,1) and consistency ICC(C,1) between both measurement systems. We used the values of each stride and average values for each participant per session separately.

The linear regression analysis was used to determine the relationship between the selected control parameter, i.e., the peak knee flexion torque, and spatiotemporal metrics of interest. For each metric, the coefficient of determination was extracted using the Pearson’s correlation and the corresponding *p*-value was computed. We defined mild, moderate, and strong relationships as having *r* values ranging 0.25–0.49, 0.50–0.69, and 0.70–1.0, respectively. The F-tests were used to evaluate statistical significance. The level of significance was set to *p* < 0.05. We also analyzed the results in relation to the level of gait function, which was quantified as the gait speed during the 10MWT at the baseline, i.e., lower gait speed was associated with a lower level of gait function (Salbach et al., 2004).

To validate offline the performance of the regression models that related the variation of the selected spatiotemporal metric obtained from the IMUs data with the peak knee flexion torque parameter, the MAE and Pearson’s determination coefficient (*R* ^2^) were used as measures of error dispersion between the actual and predicted values. Additionally, the results were also analyzed in relation to the gait speed measured during the 10MWT at the baseline.

The corrected Akaike information criterion (AICc) was the outcome metric used to determine the most suitable neural network architecture for this application (Hurvich and Tsai, 1989). AICc compared the performance and complexity of the neural network and the linear regression model.

For the neural network, a hyperparameter optimization was performed via grid search to find the model that was best adapted to the available data. The combinations tested were three activation functions, i.e., Rectified Linear Unit (ReLU), tanh, and sigmoid, and one-to-three hidden layers with different nodes, i.e., 5, 10, 15, 20, 25, and 30. The selected configuration of the neural network had one layer and five hidden nodes with ReLU as the activation function.

## 3 Results

### 3.1 Estimation of spatiotemporal metrics for clinical gait analysis

**FIGURE 3:**
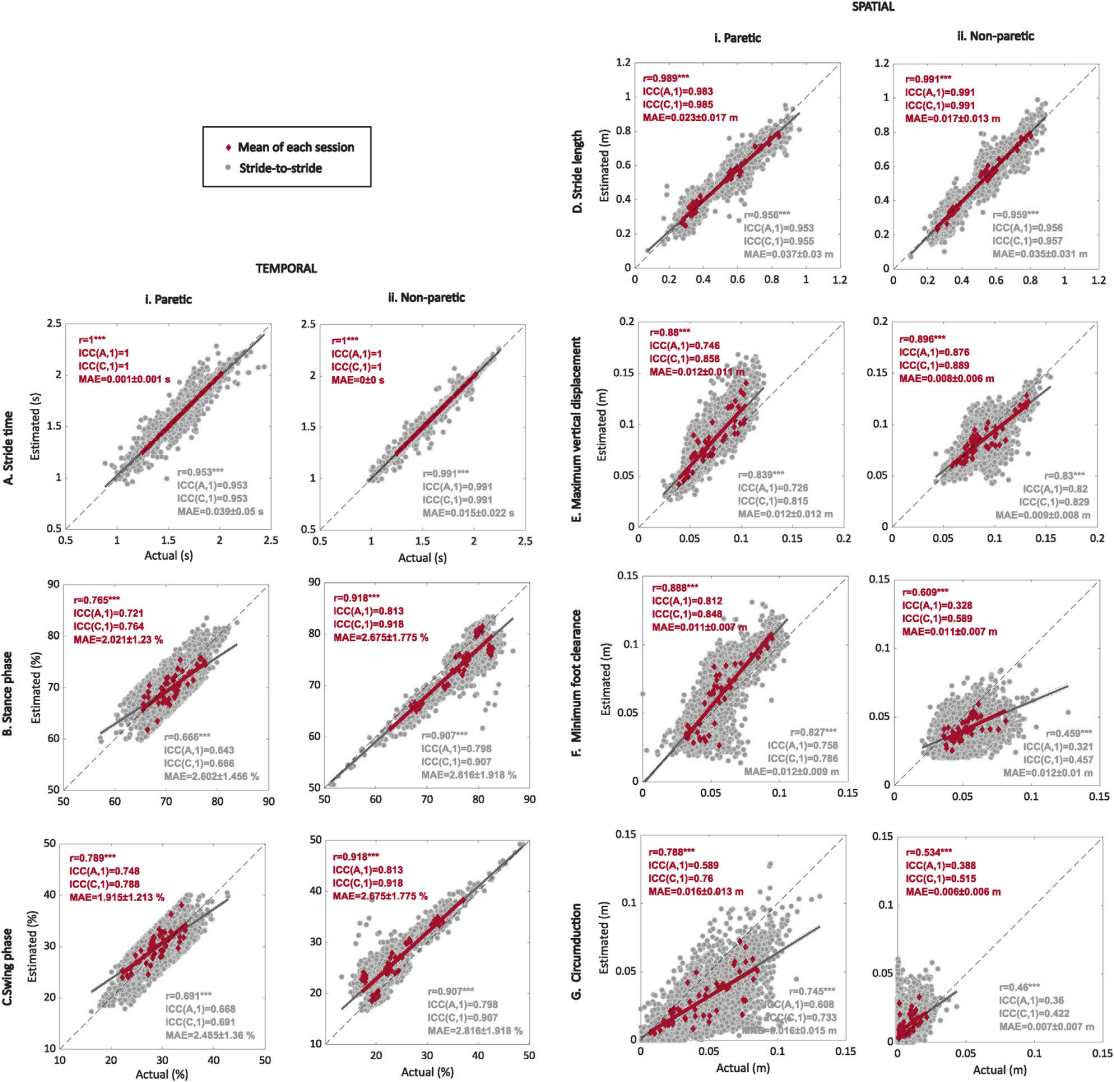
Regression plots for the temporal and spatial gait metrics comparing the estimated values obtained with the IMU-based system with the ground truth values obtained with the optical motion capture system. Average (red) and stride-to-stride (gray) estimations of the stride time **(A)**, stance **(B)** and swing **(C)** phases, stride length **(D)**, maximum vertical displacement **(E)**, minimum foot clearance **(F)**, and circumduction **(G)** for the paretic (i) and non-paretic (ii) sides. Regression plots show Pearson’s correlation *r* between the actual values obtained with the optical motion-capture system and estimated values using the shank-worn IMU-based algorithm, with *** indicating *p* < 0.001. Plots show linear fit (gray and red) and identity lines (black dashed) representing perfect estimation. For the statistical analysis, we also included the inter-class correlation coefficients for both agreement ICC(A,1) and consistency ICC(C,1), along with the mean absolute error (MAE).

The estimated averaged values of the gait temporal parameters using the IMUs for each session showed a strong correlation (0.76 < *r* < 0.99, *p* < 0.001) and inter-rater reliability both in terms of agreement and consistency [0.72 < ICC(A,1) < 1.00; 0.76 < ICC(C,1) < 1.00] (see Figure 3 and Table 2) with the metrics obtained with the optical motion capture system. The most robust estimations were found for the stride time of the paretic and non-paretic sides [*r* = 1; ICC(A,1) = 1; ICC(C,1) = 1], with the mean estimation errors lower than 0.002 s (see Figure 3A). The lowest correlation indexes were found for the stance duration [*r* = 0.765; ICC(A,1) = 0.721; ICC(C,1) = 0.764; see Figure 3Bi] and swing duration [*r* = 0.789; ICC(A,1) = 0.748; ICC(C,1) = 0.788; see Figure 3Ci] of the paretic side, with an estimated mean error lower than 3% of the gait cycle.

**TABLE 2:** Summary of mean ± std (range) of the optical motion capture (ground truth) and inertial measurement unit (IMU) for each metric along with the mean error between the ground truth motion capture and IMU-estimated values.

	Ground truth value		IMU value		Mean error	
	P	NP	P	NP	P	NP
CD (m)	0.043 ± 0.022 (0.078)	0.006 ± 0.005 (0.020)	0.028 ± 0.017 (0.067)	0.011 ± 0.007 (0.029)	0.015 ± 0.013 (0.064)	−0.005 ± 0.006 (0.030)
MVD (m)	0.071 ± 0.018 (0.065)	0.088 ± 0.020 (0.074)	0.083 ± 0.023 (0.098)	0.085 ± 0.017 (0.068)	−0.011 ± 0.011 (0.041)	0.003 ± 0.009 (0.040)
FC (m)	0.056 ± 0.017 (0.063)	0.051 ± 0.009 (0.045)	0.062 ± 0.023 (0.078)	0.040 ± 0.007 (0.033)	−0.0062 ± 0.011 (0.061)	0.010 ± 0.007 (0.040)
SL (m)	0.483 ± 0.160 (0.566)	0.470 ± 0.153 (0.547)	0.472 ± 0.147 (0.523)	0.465 ± 0.158 (0.545)	0.011 ± 0.026 (0.113)	0.004 ± 0.021 (0.099)
StP (%)	71.22 ± 3.12 (12.18)	76.55 ± 4.93 (19.89)	70.12 ± 3.00 (13.98)	74.03 ± 4.907 (19.81)	1.10 ± 2.10 (8.50)	2.52 ± 1.99 (7.56)
SwP (%)	28.75 ± 3.13 (12.31)	23.45 ± 4.93 (19.89)	29.81 ± 3.06 (14.13)	25.97 ± 4.907 (19.81)	−1.05 ± 2.01 (8.00)	−2.52 ± 1.99 (7.56)
ST (s)	1.62 ± 0.19 (0.76)	1.62 ± 0.19 (0.76)	1.62 ± 0.19 (0.76)	1.62 ± 0.19 (0.76)	0.00 ± 0.00 (0.00)	0.00 ± 0.00 (0.00)

P, paretic; NP, non-paretic; CD, circumduction; MVD, maximum vertical displacement; FC, foot clearance; SL, stride length; StP, stance phase; SwP, swing phase; ST, stride time.

The estimation of temporal metrics at a stride-to-stride level using the IMU-based system showed a moderate to strong correlation (0.66 < *r* < 0.99) together with inter-rater reliability agreement [0.64 < ICC(A,1) < 0.99] and consistency [0.66 < ICC(C,1) < 0.99] with the metrics obtained using the optical motion capture system. The stride time for the paretic [*r* = 0.953; ICC(A,1) = 0.953; ICC(C,1) = 0.953; see Figure 3Ai] and non-paretic [*r* = 0.991; ICC(A,1) = 0.991; ICC(C,1) = 0.991; see Figure 3Aii] sides showed the highest correlation coefficients, but the mean absolute errors for the paretic and non-paretic sides increased up to 0.039 s and 0.015 s, respectively. The lowest correlation coefficients were found for the duration of the stance [*r* = 0.666; ICC(A,1) = 0.643; ICC(C,1) = 0.666] and swing [*r* = 0.691; ICC(A,1) = 0.668; ICC(C,1) = 0.691] phases, with the mean estimation errors being lower than 3% of the gait cycle (see Figures 3B–C).

Averaged spatial metrics obtained with the IMU-based system showed a moderate to strong correlation (*r* = [0.53, 0.99], *p* < 0.01) and a poor to strong agreement [0.33 < ICC(A,1) < 0.99] and consistency [0.51 < ICC(C,1) < 0.99] with the metrics obtained with the optical motion capture system (see Figure 3). The highest correlation indexes were found for the stride length of the paretic [*r* = 0.989; ICC(A,1) = 0.983; ICC(C,1) = 0.985] and non-paretic [*r* = 0.991; ICC(A,1) = 0.991; ICC(C,1) = 0.991] sides, with a mean estimation error lower than 0.023 m (see Figure 3D). The lowest correlation indexes were found for the non-paretic minimum foot clearance [*r* = 0.609; ICC(A,1) = 0.328; ICC(C,1) = 0.589; see Figure 3Fii] and circumduction [*r* = 0.534; ICC(A,1) = 0.388; ICC(C,1) = 0.515; see Figure 3Gii].

The stride-to-stride estimation of the spatial metrics did not remarkably affect the strength of the correlation (*r* = [0.46, 0.96], *p* < 0.001) or the inter-rater reliability agreement [0.32 < ICC(A,1) < 0.96] and consistency [0.46 < ICC(C,1) < 0.96] when compared to the estimation using the averaged values (see Figure 3).

**FIGURE 4:**
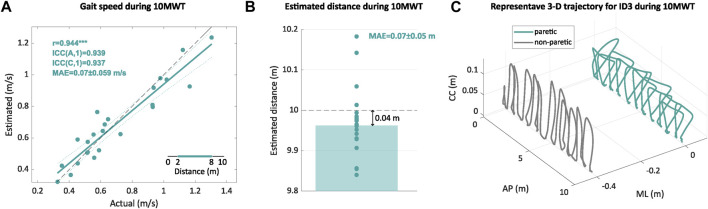
Estimated gait speed and distance for the 10MWT. **(A)** Regression plot comparing the estimated gait speed with the IMU-based system and the actual value for each trial. **(B)** Bar plot with the estimated distance covered during the 10MWT with the IMU-based system for each trial (circles) and the mean of all the trials (bar). **(C)** Example of a continuous gait trajectory obtained with the IMU-based system for paretic and non-paretic sides during the 10MWT. The regression plot shows the Pearson’s correlation *r* between actual values and the estimates obtained with the IMU-based system, with *** indicating *p* < 0.001. The regression plot shows the linear fit (green) and identity lines (black dashed) representing perfect estimation. For the statistical analysis, we also included the inter-class correlation coefficients for both agreement ICC(A,1) and consistency ICC(C,1), along with the mean absolute error (MAE). In the axes, CC means the craniocaudal direction, AP means the anteroposterior direction, and ML means the mediolateral direction.

The estimated overground gait speed obtained with the IMU-based system for the 10MWT showed a strong correlation [*r* = 0.944, *p* < 0.001], with a strong agreement [ICC(A,1) = 0.94] and consistency [ICC(C,1) = 0.94], with the gait speed, which was obtained with the digital stopwatch (see Figure 4A). The MAE for the estimation of gait speed and distance covered was 0.07 ± 0.059 m/s and 0.07 ± 0.050 m, respectively (see Figure 4B). As an example, Figure 4C shows continuous gait trajectories estimated by the proposed method during the 10MWT (participant ID3).

### 3.2 Relationship between estimated spatiotemporal metrics and peak knee flexion torque

**FIGURE 5:**
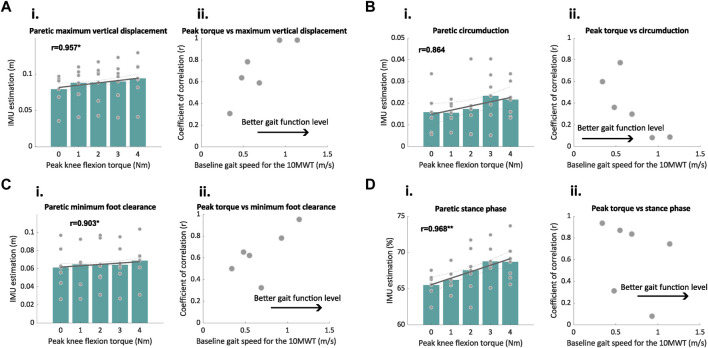
Relationships between the spatiotemporal metrics of interest estimated by the IMUs and peak knee flexion torque. **(A–D)** Correlation plots between peak knee flexion torque and the spatiotemporal outcomes estimated by the IMUs (i), and the relationship between the coefficient of correlation and level of gait function of the participants measured with the gait speed during the 10MWT at baseline (ii). The gray lines in subplot (i) depict the best fit line; *r* values are the coefficients of correlation; the green bar plots represent the average value of all participants for the IMU-estimated metrics, and the gray dots represent the average of each participant. The gray points in subplot (ii) represent the correlation coefficients found for each metric and participant in relation to the motor function of each participant (measured as the gait speed at baseline). *p < 0.05, **p < 0.01.

Paretic maximum vertical displacement (*r* = 0.96, *p* = 0.010; see Figure 5Ai), minimum foot clearance (*r* = 0.90, *p* = 0.015; see Figure 5Ci), and circumduction (*r* = 0.86, *p* = 0.058; see Figure 5Bi) had the highest significant positive correlations with the peak knee flexion torque parameter (see Table 3). Regarding the temporal metrics of the paretic side, there were significant strong correlations for the duration of the stance (*r* = 0.97, *p* = 0.006; see Figure 5Di) and swing (*r* = −0.97, *p* = 0.007; see Table 3) phases. Low to moderate Pearson’s correlation coefficient values (0.06 < *r* < 0.68) were observed for the spatiotemporal metrics for the non-paretic side (see Table 3). The mean and standard deviation of each metric for each torque level is given in Tables 4 and 5.

**TABLE 3:** Relationship between the spatiotemporal metrics of interest estimated by the IMUs and peak knee flexion torque per participant.

Participant	Coefficient of correlation (r) with the peak knee flexion torque													
	CD		MVD		FC		SL		StP		SwP		ST	
	P	NP	P	NP	P	NP	P	NP	P	NP	P	NP	P	NP
ID 1	0.95	0.01	0.98	−0.51	0.95	0.67	0.68	0.63	0.74	0.34	−0.74	−0.34	0.73	0.71
ID 2	0.50	0.54	0.30	0.70	0.50	−0.01	0.74	0.71	0.94	0.65	−0.94	0.83	0.75	0.76
ID 3	0.32	0.01	0.59	0.77	0.20	0.40	−0.12	−0.62	0.84	0.44	−0.84	−0.44	0.13	0.15
ID 4	0.62	0.55	0.78	0.72	0.62	0.61	−0.30	−0.12	0.87	0.87	−0.88	−0.23	−0.86	−0.85
ID 5	0.78	0.00	0.98	0.02	0.78	0.05	−0.43	−0.34	0.08	0.30	−0.08	0.30	−0.44	−0.44
ID 6	0.65	0.35	0.63	−0.36	0.48	0.36	−0.35	−0.15	0.31	0.09	−0.31	0.09	−0.21	−0.21
**All**	0.86	0.66	0.96*	0.46	0.90*	0.68	0.32	0.46	0.97**	0.62	−0.97**	0.06	0.66	0.68

P, paretic; NP, non-paretic; CD, circumduction; MVD, maximum vertical displacement; FC, foot clearance; SL, stride length; StP, stance phase; SwP, swing phase; ST, stride time. *p < 0.05, **p < 0.01.

The values in bold indicate statistical significance.

**TABLE 4:** Mean ± std of the spatial metrics of interest for each peak knee flexion torque level.

Peak knee flexion torque (Nm)	CD (m)		MVD (m)		FC (m)		SL (m)	
	P	NP	P	NP	P	NP	P	NP
0	0.015 ± 0.010	0.011 ± 0.004	0.079 ± 0.023	0.087 ± 0.017	0.059 ± 0.022	0.038 ± 0.012	0.499 ± 0.214	0.485 ± 0.222
1	0.015 ± 0.005	0.012 ± 0.007	0.084 ± 0.026	0.087 ± 0.017	0.062 ± 0.024	0.040 ± 0.008	0.502 ± 0.232	0.495 ± 0.232
2	0.017 ± 0.012	0.011 ± 0.006	0.086 ± 0.028	0.088 ± 0.020	0.062 ± 0.023	0.040 ± 0.007	0.515 ± 0.214	0.505 ± 0.224
3	0.023 ± 0.012	0.013 ± 0.008	0.089 ± 0.029	0.086 ± 0.018	0.064 ± 0.023	0.039 ± 0.008	0.508 ± 0.219	0.493 ± 0.235
4	0.021 ± 0.0087	0.012 ± 0.004	0.090 ± 0.031	0.089 ± 0.018	0.066 ± 0.025	0.041 ± 0.008	0.502 ± 0.215	0.496 ± 0.224

P, paretic; NP, non-paretic; CD, circumduction; MVD, maximum vertical displacement; FC, foot clearance; SL, stride length.

**TABLE 5:** Mean ± std of the temporal metrics of interest for each peak knee flexion torque level.

Peak knee flexion torque (Nm)	StP (%)		SwP (%)		ST (s)	
	P	NP	P	NP	P	NP
0	65.49 ± 1.79	72.74 ± 4.67	34.39 ± 1.63	27.14 ± 4.83	1.49 ± 0.23	1.49 ± 0.23
1	66.20 ± 1.65	72.74 ± 4.95	33.76 ± 1.65	27.21 ± 4.99	1.50 ± 0.23	1.50 ± 0.24
2	67.51 ± 3.41	72.92 ± 4.87	32.40 ± 3.26	27.01 ± 4.98	1.54 ± 0.26	1.54 ± 0.26
3	68.75 ± 2.48	72.85 ± 4.81	31.24 ± 2.48	27.14 ± 4.80	1.52 ± 0.26	1.52 ± 0.26
4	68.69 ± 2.97	72.83 ± 4.69	31.30 ± 2.97	27.16 ± 4.69	1.52 ± 0.25	1.53 ± 0.25

P, paretic; NP, non-paretic; StP, stance phase; SwP, swing phase; ST, stride time.

To understand how the gait function level of the participants affected the strength of the correlations, we also analyzed the relationship between the baseline gait speed of the participants during the 10MWT and correlation coefficients (see Figures 5A–Dii; Table 3). At the participant level, the results showed a large deviation in the coefficient of correlation among participants depending on their gait function level. We observed that for paretic maximum vertical displacement and minimum foot clearance, the coefficient of correlation increased for the participants with a higher baseline speed, while for circumduction, the coefficient of correlation decreased for the participants with a higher baseline speed (see Figures 5A–Cii).

### 3.3 Offline validation of using IMU-based estimations of spatiotemporal metrics for design of adaptive control strategies

**FIGURE 6:**
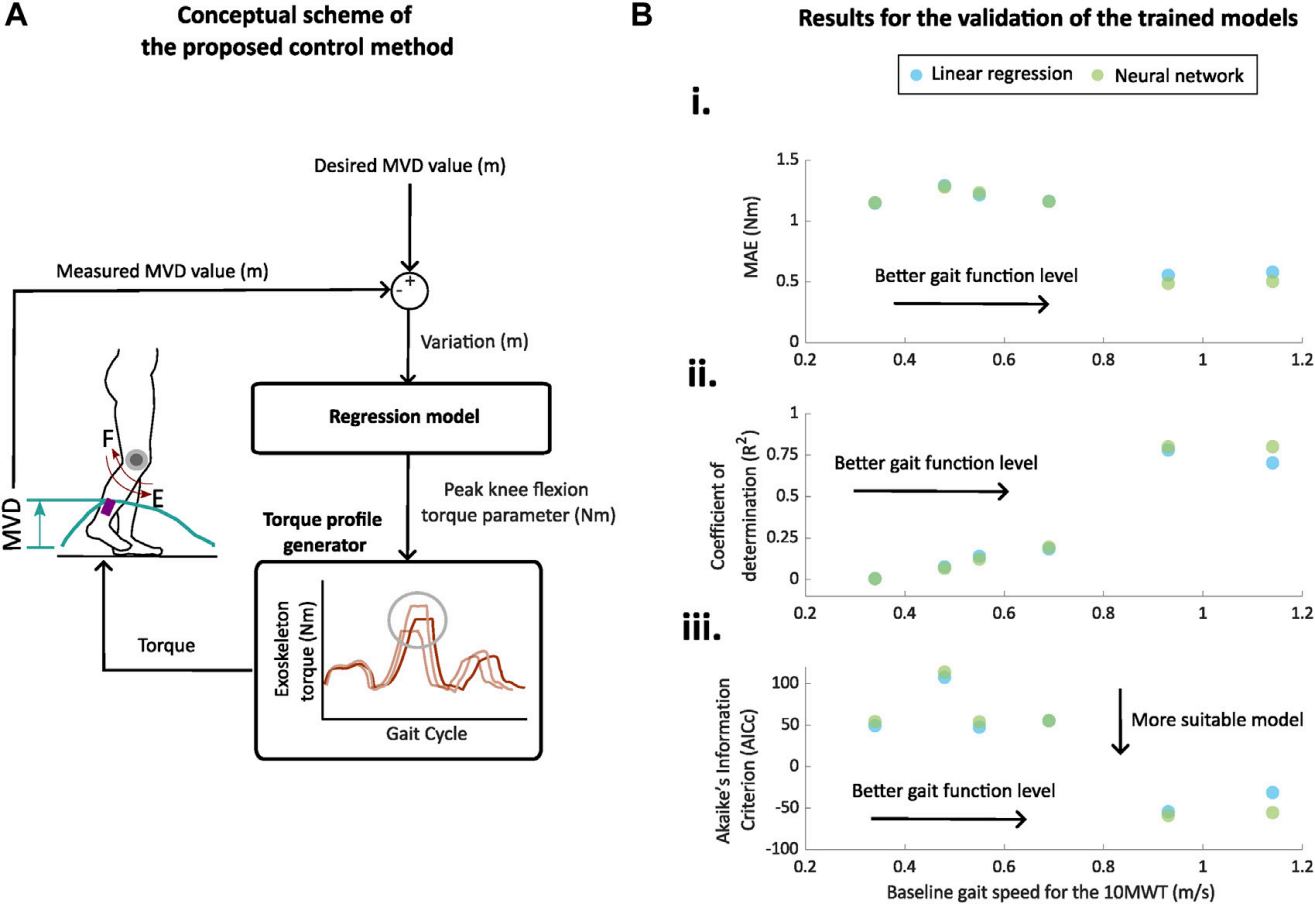
Proposed control method and offline validation results. **(A)** Conceptual diagram of the proposed adaptive control strategy for a knee exoskeleton. Maximum vertical displacement (MVD) is estimated from previous strides using the data from IMUs, and a regression model (neural network in this example) is used to generate a new value for the peak knee flexion torque parameter. **(B)** (i) Mean absolute errors (MAEs), (ii) coefficients of determination (R^2^), and (iii) corrected Akaike Information Criterion (AICc) for the prediction of the peak knee flexion torque of the trained linear regression (blue) and neural network (green) models in relation to the gait function of the participants. The gait function was associated with the gait speed during the 10MWT at baseline (Salbach et al., 2004).

From the previous results, we identified that the paretic maximum vertical displacement had the strongest correlation when compared with the optical system, and also a high correlation with the exoskeleton parameter that controls the peak knee flexion torque (see Figure 6A).

The results for the evaluation of the linear regression model and neural network model with 20% of the steps in relation to the gait speed measured during the 10MWT at baseline are presented in Figure 6B and Table 6. Predictions for peak knee flexion torque were more accurate for participants with a higher gait speed during the 10MWT, i.e., better gait function (*R* ^2^ = 0.70–0.78; MAE = 0.55–0.58 Nm). These participants also had the highest correlation index between the maximum vertical displacement and the peak knee flexion torque (*r* = 0.96; see Figure 5Bi). The lowest levels of the determination coefficient (*R* ^2^ = 0.00–0.18) and highest MAEs (MAE = 1.15–1.29 Nm) were found for the participants with worse gait function. These participants had lower levels of correlation indexes between the maximum vertical displacement and peak knee flexion torque (*r* = 0.3–0.78; see Figure 5Bii).

**TABLE 6:** Results of the offline validation for the linear regression and neural network.

Participant	Linear regression			Neural network		
	MAE (Nm)	*R* ^2^	AICc	MAE (Nm)	*R* ^2^	AICc
ID 1	0.58	0.70	−31.14	0.50	0.80	−55.36
ID 2	1.15	0.00	49.52	1.15	0.00	54.36
ID 3	1.16	0.18	55.49	1.16	0.19	55.49
ID 4	1.21	0.14	47.75	1.23	0.12	53.99
ID 5	0.55	0.78	−54.33	0.48	0.80	−58.81
ID 6	1.29	0.08	107.76	1.28	0.07	113.92

MAE, mean absolute error; *R* ^2^, coefficient of determination; AICc, Akaike Information Criterion.

The neural network model better predicted the peak knee flexion torque in terms of MAE and *R* ^2^ than the linear regression model (see Figure 6B). The predictions of peak knee torque for the participants with a better gait function (i.e., higher velocities during the baseline 10MWT) had high coefficients of determination and low MAE (*R* ^2^ = 0.78–0.80; MAE = 0.48–0.50 Nm). For these participants, the complexity added to the neural network with respect to the linear model was justified in terms of performance, i.e., lower values for the AICc with the neural network (neural network vs. linear regression = ID1: −55 vs. −31 and ID5: −58 vs. −54). For participants with worse gait function (i.e., lower gait speed during the baseline session), the prediction of the peak torque was considerably worse as also observed with the linear regression model (*R* ^2^ = 0.00–0.19; MAE = 1.15–1.28 Nm). For this group of participants, the complexity added to the neural network with respect to the linear model was not justified in terms of performance, i.e., higher values for the AICc with the neural network.

## 4 Discussion

In this work, we validated the feasibility of using shank-worn IMUs for clinical gait analysis after stroke and evaluated their preliminary applicability in designing an automatic and adaptive controller for a knee exoskeleton. The main contribution of the present study relies in the stride-to-stride estimation of spatiotemporal metrics using two shank-worn IMUs to design adaptive control laws for knee exoskeletons after stroke. First, we validated a gait monitoring algorithm capable of estimating the spatiotemporal metrics using two shank-worn IMUs with data from six participants after stroke when walking on a treadmill and overground. Second, we analyzed the relationship between the spatiotemporal metrics obtained from the IMU data and the parameter that sets the peak knee flexion torque of a unilateral knee exoskeleton (ABLE-KS). Finally, we designed and validated offline a set of control laws using two regression models, linear regression and neural network, to automatically adapt the peak knee flexion torque parameter based on the maximum vertical displacement. We selected this metric as it had the highest reliability in the estimation and correlation with the control parameter.

### 4.1 Reliability of estimating gait spatiotemporal metrics using shank-worn IMUs after stroke

We can confirm that IMUs placed at the shanks of the paretic and non-paretic sides can provide valuable estimates of spatiotemporal metrics for gait analysis in post-stroke individuals. However, it is important to consider that the accuracy of estimation is highly related to the level of impairment and the location of the IMU sensors (Picerno, 2017; Carcreff et al., 2018; Niswander et al., 2020).

In the case of the stride time, our estimation (*r* = 1; MAE = 0.001 s) was better than the one reported in other studies that placed the IMUs at the thighs (MAE = 0.028 s) (Arumukhom Revi et al., 2021) or lower back (*r* = 0.965) (Parisi et al., 2016). Our results for the stance and swing ratios (MAE = [1.91–2.67] %; *r* = [0.76–0.92]) were similar to those of Laidig et al. (2021) where an IMU was placed at the foot (MAE = 1.43%; *r* = 0.89), and to those of Parisi et al. (2016) where one IMU was placed at the lower back (*r* = [0.86–0.91]).

Our estimation quality for the stride length (MAE = 0.023ii *r* = 0.99) was aligned with other studies that used IMUs placed at the feet (MAE = [0.001, 0.038] m; *r* > 0.99) (Arens et al., 2021; Laidig et al., 2021) and better than the studies that placed the IMUs at the thigh and shank (MAE = 0.035 m) or only shanks (*r* = [0.81, 0.942]) (Visi et al., 2017; Hendriks et al., 2022). Contrarily, a study that used two IMUs placed at both feet obtained better estimation results than the ones reported here for the circumduction metric [*r* = 0.534 vs. *r* = 0.920; MAE = 0.006 m vs. MAE = 0.002 m; ICC(C,1) = 0.515 vs. ICC(C,1) = 0.852; ICC(A,1) = 0.388 vs. ICC(A,1) = 0.868] and the maximum vertical displacement [*r* = 0.88 vs. *r* = 0.912; MAE = 0.012 m vs. MAE = 0.006 m; ICC(C,1) = 0.858 vs. ICC(C,1) = 0.912; ICC(A,1) = 0.746 vs. ICC(A,1) = 0.847] in individuals after stroke (Arens et al., 2021). However, in the cited study, authors only had analyzed 9-48 strides per participants, while we have analyzed 530–650. This difference in the number of analyzed strides might explain the difference in the quality of estimation, as analyzing a lower number of strides might lead to less data variability and to consequently more accurate estimations. The estimation of the paretic minimum foot clearance [*r* = 0.89; MAE = 0.01 m; ICC(A,1) = 0.812; ICC(C,1) = 0.848] was found to be robust enough to be used in a clinical environment (Al Bochi et al., 2021). However, we have not found any other study that validated the estimation of this metric using IMUs in participants after stroke (Yang et al., 2013; Parisi et al., 2016; Visi et al., 2017; Wang et al., 2018; Feuvrier et al., 2020; Arens et al., 2021; Arumukhom Revi et al., 2021; Laidig et al., 2021; Hendriks et al., 2022) or with other brain injuries (Bourgeois et al., 2014; Sijobert et al., 2015; Moon et al., 2017; Behboodi et al., 2019).

The estimation of overground gait speed during the 10MWT (MAE = 0.07 m/s) was good enough to detect minimal clinically important differences (0.14 m/s) for post-stroke gait analysis (Livolsi et al., 2022; Shin et al., 2022). Our results were slightly better than the ones reported in other studies that estimated the same metrics with shank-worn (*r* = 0.94 vs. *r* = 0.93; MAE = 0.07 m/s vs. MAE = 0.09 m/s) (Yang et al., 2013; Hendriks et al., 2022) and foot-worn (MAE = 0.07 m/s vs. MAE = 0.1 m/s) (Feuvrier et al., 2020) IMUs.

At the stride level, the estimation of most of the spatiotemporal metrics of interest was accurate enough to consider these metrics as candidates for the application of real-time adaptive control due to the high value of the correlation coefficients. We found that the estimations of the non-paretic minimum foot clearance (see Figure 3Fii) and circumduction (see Figure 3Gii) were not robust enough to be considered as candidate metrics to adapt the control action of an exoskeleton for people after stroke. The reason is that most of the actual values of these metrics might be out of the range of precision of the IMU sensor and sampling frequencies used in this study.

### 4.2 Relationship between peak knee flexion torque and spatiotemporal outcomes estimated with shank-worn IMUs

At a group level, strong correlations were found for the peak knee flexion torque and the estimation of circumduction, minimum foot clearance, maximum vertical displacement, and stance and swing phases of the paretic side. Our findings agree with those of Sulzer et al. (2010) and Fricke et al. (2020b), where the authors also found positive relationships between peak knee flexion torque and step length, circumduction, minimum foot clearance, foot maximum vertical displacement, and stance time.

However, we have seen that the coefficients of correlation at a participant level presented high variations depending on the level of gait function. In the present study, we have not found any spatiotemporal metric that strongly correlated with the peak knee flexion torque for all the levels of gait function of the included participants. This finding highlights the fact that the correlation between the biomechanical descriptors and control parameters can be highly dependent on the level of gait function (Fricke et al., 2020b).

### 4.3 Can shank-worn IMUs be used to design adaptive control strategies for knee exoskeletons for post-stroke rehabilitation?

We selected the maximum vertical displacement estimated from the IMU data as the input for evaluating the performance of the proposed adaptive controller. It was the metric that presented the best accuracy against the optical motion capture system and highest correlation index with the peak knee flexion torque. As a tentative approach for future implementation, we developed and validated off-line a controller based on regression-based models, i.e., linear regression and neural network.

When comparing both models, we obtained a better estimation of the peak torque with the neural network than with the linear regression. However, despite the higher complexity and capacity of modeling embedded non-linearity between input and output variables, the performance of the neural network did not remarkably surpass the performance of the linear regression model.

Considering the predictions generated by the regression models, we consider that these models can be implemented to design adaptive control strategies that automatically tune the peak knee flexion torque based on the maximum vertical displacement estimated by IMUs for participants that have a moderate degree of gait function, i.e., gait speed higher than 0.7 m/s (Salbach et al., 2004). However, the quality of the predictions for the peak knee torque was considerably worse for the participants with worse levels of gait function, i.e., gait speed lower than 0.7 m/s. None of the models could provide good predictions of the peak torque parameter based on the maximum vertical displacement for all the participants, despite the fact that the majority of the more impaired participants had a moderate to strong correlation between these variables, i.e., *r* = 0.6–0.78. Future studies should focus on finding other biomechanical descriptors that have a higher correlation with a wider range of participants and validating more complex regression models that might better capture the population heterogeneity observed.

Our proposed approach complements other automatic methods found in the literature, which adapt control parameters based on joint kinematic errors (Fricke et al., 2020a) or normal ground reaction forces (Blaya and Herr, 2004). These methods adapt the control parameters in a discrete and sequential way, starting from a predefined level of assistance and changing the assistance level by a predefined amount at each iteration. On the contrary, the controller proposed here can adapt without predefining the initial conditions or amplitude of the change for each iteration.

Controllers that use optimization rules to get the optimal set of parameters have evolved highly in recent years (Ding et al., 2018; Díaz et al., 2022; Slade et al., 2022). However, human-in-the-loop parameter optimization is still far from being applicable for individuals with neurological impairments due to the long walking times that are required for the algorithms to converge (Nguyen et al., 2019; Siviy et al., 2020). The results and methodology of the present study can potentially be used in combination with human-in-the-loop algorithms to reach faster convergence to the optimal set of control parameters for clinical populations.

### 4.4 Limitations

The presented work has a number of limitations that can guide future work in this field. The exploratory findings presented and discussed here should be interpreted with caution due to the small and heterogeneous sample. Although the proposed method has the potential to be implemented in other clinical conditions leading to hemiplegia, the noticeable differences in the gait patterns of the participants and compensatory movements made it difficult to generalize the results. Future studies should include larger population sizes with similar gait functions to further demonstrate the feasibility of this approach.

The participants had not tested the different peak knee flexion torque levels in a random order, thus the order of the experimental conditions might have affected the results of this study. However, note that during sessions 2–5, the participants had been already trained with the different torque levels. Moreover, we believe that the variability resulting from the heterogeneity in motor function between participants most likely had a higher impact than the fixed order of the experimental conditions.

Hardware limitations were also notable in the estimation of the spatiotemporal metrics. The selection of the hardware and location of the IMUs were based on the ones used in the exoskeleton of this study. Moreover, there are other commercial IMUs that have a higher sampling frequency and accuracy, which might have provided better results.

For the selection of the control model, we could have used more complex models, i.e., deep neural networks, than the proposed regression models for the estimation of the exoskeleton torque parameter, which might have exhibited a higher performance. However, the use of these models would have required a comprehensive analysis of the tuning of the hyperparameters that was out of the scope of this feasibility study. Future work should explore other models that might better capture the heterogeneity of the post-stroke gait to adapt the exoskeleton torque and their implementation in real time.

## 5 Conclusion

We implemented and evaluated a method to estimate spatiotemporal metrics in six post-stroke participants while walking overground and on a treadmill, by using a pair of low-cost IMUs placed on the shanks. The estimations at the stride level were sufficiently reliable to apply these metrics for real-time adaptive control applications on post-stroke gait. Subsequently, we studied the relationship between the peak knee flexion torque parameter of a knee exoskeleton and spatiotemporal metrics estimated by the shank-worn IMUs. The maximum vertical displacement was the metric that had the highest correlation with the peak knee flexion torque parameter of the exoskeleton for the different gait function levels of the participants and strong correlation coefficients with the data from the optical motion-tracking system. Finally, we performed an offline validation of two machine learning models, i.e., linear regression and neural network, to adapt the peak knee flexion torque based on variations of the maximum vertical displacement estimated by the shank-worn IMUs. Although the neural network presented a better performance than the linear regression, this difference was not remarkable. Real-time implementation is still an open path for future development, but our preliminary results have demonstrated the feasibility of this approach to design adaptive control strategies for lower limb exoskeletons for people with moderate impairments in gait function due to stroke. Future work will include the comparison of control strategies that automatically adapt the control parameters *versus* the standard manual tuning.

## Data Availability

Due to patient confidentiality and proprietary information of ABLE Human Motion S.L., the datasets generated and/or analyzed during the current study are not publicly available, but they are available from the corresponding author on reasonable request with permission of the third party. Requests to access the data sets should be directed to jesus.de.miguel@upc.edu.
